# Bibliometric and Visualized Analysis of the Current Status on STING Signaling Pathway and Cancer

**DOI:** 10.1155/2022/5095176

**Published:** 2022-11-23

**Authors:** Zhongqi Lu, Qiang Fu, Jinyuan Sui, Ying Chang, Tiefeng Jin, Meihua Zhang

**Affiliations:** ^1^Department of Ultrasound Medicine, Affiliated Hospital of Yanbian University, Yanji 133000, Jilin, China; ^2^Tumor Research Center of Yanbian University, Yanji 133000, Jilin, China; ^3^Health Examination Centre, Yanbian University Hospital, Yanji 133002, Jilin, China

## Abstract

Cancer, as the second leading cause of death worldwide, has become an ongoing public health challenge and its treatment has received much attention, with immunotherapy becoming a hot research topic in recent years. The interferon gene stimulating factor (STING)-mediated signaling pathway has a “double-edged sword” role in cancer, which plays different roles in different types and stages of tumors. In this paper, we discuss the current research status, cooperation, and hotspots of STING signaling pathway in cancer from 2008–2022 using CiteSpace software based on the literature of cancer and STING signaling pathway. In addition, we predicted future research trends in this field by analysis, and the results showed that the STING signaling pathway is rapidly increasing in cancer research, and its role in tumor microenvironment and immunotherapy has become a new hot spot in current research and will continue to receive high attention.

## 1. Introduction

With continuous changes in the environment and lifestyle, cancer has become the second most life-threatening disease after cardiovascular disease. GLOBOCAN 2020 estimated that there were 19,292,789 cancer cases and 9,958,133 cancer deaths globally in 2020 [1]. Living with cancer has become a forced choice for many people. Delaying the progression of cancer, preventing the metastasis of cancer cells, and improving the quality of life of cancer patients are the unremitting pursuits of many scholars in the field of oncology. A growing number of studies suggest that tumorigenesis does not occur in isolation, that all cells in the human body live in a complex ecosystem of cells—its so-called microenvironment. All cells may interact via juxtacrine and paracrine mechanisms in the microenvironment [2]. The occurrence of cancer should be a pathological ecosystem of “ecological and evolutionary unity” in multidimensional time and space, in which cancer cells and other cells interact and coevolve in time and space [3]. With in-depth research on tumor microenvironment, immunotherapy has emerged as a cancer treatment that has received much attention after surgery and radiotherapy [4]. It was named the most important scientific breakthrough of the year in 2013 by the journal Science, and has been widely studied by scholars recently with good progress [5].

The cGAS-cGAMP-STING-mediated signaling pathway of type I interferon (IFN-1) is an important discovery in the field of innate immunity, which plays a crucial role in various tissues, organs, and diseases [6]. STING is a key signaling adapter protein for DNA sensing pathways, located in the endoplasmic reticulum (ER) at rest. The ER membrane provides a structural platform for activating the IFN-1 response [7]. As a DNA sensor, cyclic GMP-AMP synthetase (cGAS) recognizes cytoplasmic DNA from exotic microorganisms and mitochondrial DNA from damaged genomes when the body is invaded by inflammation and tumors [8]. cGAS binds to a double-stranded deoxyribonucleic acid (dsDNA) and converts ATP and GTP into the second messenger 2′ 3′-cGAMP, which binds and activates STING located in the endoplasmic reticulum. Then, STING translocates to the Golgi apparatus, recruiting IRF3 and NF-*κ*B via TBK1 and IKK, respectively. IRF3 and NF-*κ*B translocate into the nucleus and drive the expression of IFN and cytokines [8, 9]. Chromosomal instability (CIN) is a hallmark of cancer caused by ongoing errors in chromosome segregation during mitosis [10]. Errors in chromosome segregation produce a large number of micronuclei, which rupture and spill genomic DNA into the cytoplasm, resulting in activation of the cGAS-STING cytoplasmic DNA sensing pathway and downstream nonclassical NF-kappa B signaling [11]. CIN in cancer cells has been reported to activate the cGAS–STING innate immunity pathway via micronuclei formation, thereby influencing tumor immunity and tumor progression, inducing sequential chromosomal segregation errors that promote cell invasion and metastasis in a STING-dependent manner [10, 11].

CiteSpace software is an information, visualization, and analysis software which can visually display the development process and structural relationship of scientific knowledge. The visualized knowledge graphs are divided into different segments according to time, with forming a visual network map. It can present the trends of a research field and provide a reference for deeper exploration of the research hotspots and development frontiers in the field [12].

Based on the bibliometric approach, this paper presents a comprehensive visual analysis of the literature related to the STING signaling pathway and cancer in the Web of Science database using CiteSpace software [12, 13] and discusses the current status, research hotspots, and development trends of the STING signaling pathway on cancer progression, aiming to provide meaningful references for researchers.

## 2. Materials and Methods

### 2.1. Data Collection

The data used in this paper were obtained from the Web of Science database published by the Institute for Scientific Information (ISI). All the databases in the Web of Science Core Collection database were used as sources. We have searched for the articles containing the subject terms “STING” and “Cancer” in the journals from 2008 to 2022, with 1670 papers searched in the Web of Science Core Collection Literature [14].

### 2.2. Methods

In this paper, 1670 Web of Science Core Collection documents were exported from the Web of Science database in the form of “plain text,” with the “full records and cited references” in the exported document records as the source data to be processed. CiteSpace 5.8.R3 was used to analyze the country, institution, author, reference, and keywords in the core collection as nodes for co-occurrence analysis, cluster analysis, Timezone View, and Timeline View, setting the time span to 2008–2022. The time slice was set to 1 year, and the sources of subject terms were selected as title, abstract, author keywords (DE), keywords plus (ID), and pathfinder, and pruning sliced networks were selected as the pruning method. Then, the software was run for analysis [15].

The nodes in the map represent keywords and core citations, respectively; the size of the circle of the node represents the frequency; the larger the diameter of the circle, the higher the frequency of the node; the colour scale of the circle represents the different years of the content; the width of the circle represents the frequency of the content in different years; the line between the nodes represents the co-occurrence frequency; the thicker the line, the higher the co-occurrence frequency and the closer the relationship; the colour scale of the node and the line represents the year of publication of the corresponding literature [16].

## 3. Results

### 3.1. Annual Growth Trend of Publications

**Figure 1:**
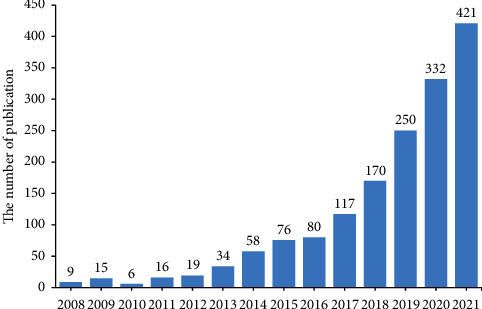
The trend of increase in publications and annual distribution between 2008 and 2022.

By searching the WoSCC database, a total of 1670 papers on STING signaling pathways and cancer were published in 2008–2022. As shown in Figure 1, in terms of temporal distribution, fewer studies of STING signaling pathways and cancer were reported between 2008 and 2016 (Figure 1). Since 2017, the number of publications increased rapidly, accounting for 81.2% of the total and peaked at 421 in 2021 (Figure 1). These data fully demonstrate that research related to STING signaling pathway and cancer have been widely concerned, and the attention continues to increase, becoming the focus of research in recent years. Based on the trend, it can be predicted that relevant research will not decrease in the coming years and will continue to grow.

### 3.2. Countries/Regions and Institutions Analysis

**Figure 2:**
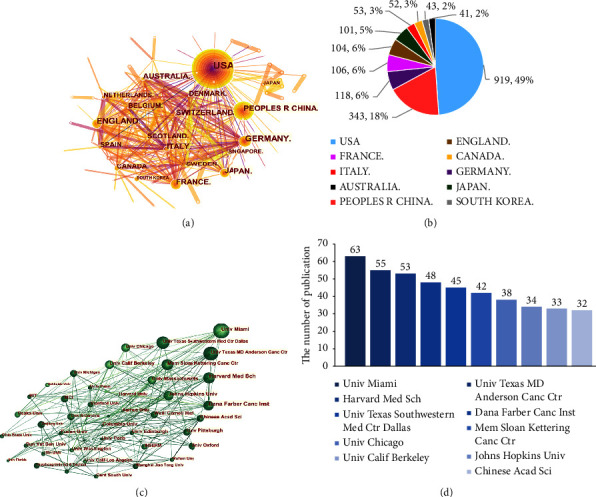
Analysis of countries/regions and institutions engaged in the research on STING signaling pathway and cancer. (a) A network map showing countries/regions involved in the research on STING signaling pathway and cancer. (b) The top 10 most productive countries/regions. (c) A network map showing institutions involved in the research on STING signaling pathway and cancer. (d) The top 10 institutions involved in the research on STING signaling pathway and cancer.

The CiteSpace software was used to conduct statistical analysis of the countries/regions issuing the documents and their cooperation networks, and create a co-occurrence map to reflect the major countries/regions in the field and their collaborative relationships (Figure 2(a)), with a density of 0.0556, 123 nodes, 417 connecting lines, and a time interval of 1 year. A total of 1670 articles came from 123 countries. It can be seen that there were a wide range of collaborations between many countries/regions in Figure 2(a), and it is notable that the USA have the most collaborations with the rest of the world. The frequency distribution of collaborations is shown in Figure 2(b), with the top 3 countries/regions being the USA (*n* = 919, 49%), Peoples R China (*n* = 343, 18%), and Germany (*n* = 118, 6%).

Figure 2(c) shows the collaboration network of research institutions across countries, with 406 organizations contributing articles. The top nine institutions are from the United States, including the University of Miami, The University of Texas MD Anderson Cancer Center, and Harvard Medical School, the top three and most collaborative institutions worldwide. These 10 institutions collaborated on 433 manuscripts (Figure 2(d)). The Chinese Academy of Sciences is the leading research institution in China engaged in research on STING signaling pathway and cancer. There are other main institutions in China, including Fudan University, Shanghai Jiao Tong University, Huazhong University of Sciences and Technology, and Capital Medical University. These scientific research teams have collaborative relationships with each other, but they lack collaboration with foreign institutions. Overall, the research on STING signaling pathway and cancer are deficient in China, indirectly suggesting that Chinese scholars need to further develop their research in this area.

### 3.3. Authors and Cocited Authors

**Figure 3:**
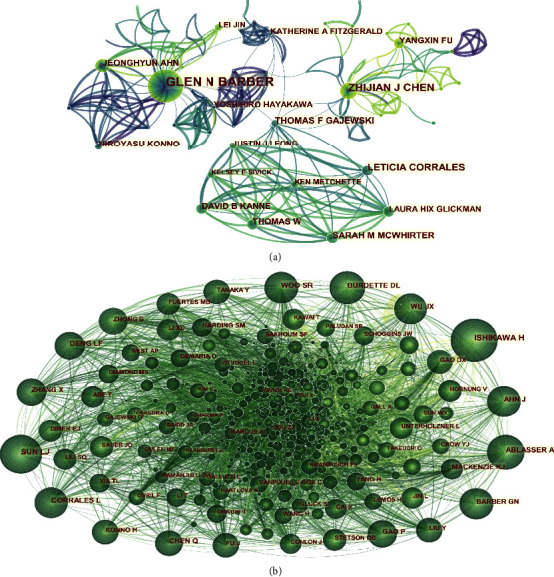
CiteSpace network of authors and cocited authors in the field of STING signaling pathway and cancer research. (a) Each node represents one author. The size of the node is positively correlated with cited counts of the authors, and links between the two circles represent a collaboration between the two authors on the same article. Line thickness is positively correlated with the frequency of collaborations. (b) Each node represents a reference. The size of the node is positively correlated with the frequency of citations, and links between the two circles represent two references that were cited in the same article.

The CiteSpace software is used to conduct statistical analysis of the core authors and their cooperative networks and create a co-occurrence map to reflect the core researchers, teams, and their collaborations in the field (Figure 3(a)), with a density of 0.0081, 571 nodes, 1320 connections, and a time slice of 1 year. It can be seen from the authors' collaboration network that there are currently 571 researchers involved in the research on STING signaling pathways and cancer. Many teams have been formed, with mutual collaborations among them, of which there are three larger ones. According to Table 1, we know that the top three authors with the most publications are Glen N Barber, Zhijian J Chen, and Leticia Corrales. Glen N Barber has been working on the research of STING signaling pathway and cancer since 2008 and has been engaged in this area with a considerable number of research results. He has collaborated with many scholars, such as Jeonghyun Ahn, Lei Jin, and Yoshihihiro Hayakawa and has become the central hub of this research system. Zhijian J Chen, the second most published scholar in China with 23 publications, is the core member of the Chinese team working on the research of STING signaling pathway and cancer. He has collaborated with most Chinese scholars, especially with Xiaodong Li, Tuo Li, Hua Wang, and Daqi Tu. It can be seen that Chinese scholars engaged in this research have formed a collaborative network system, with close cooperations. It can be predicted that the Chinese research team on STING signaling pathway and cancer will be stronger and more achievements will be made in this area. The results of statistical analysis also demonstrate that there are more intrateam collaborations in this field, but few interteam collaborations, and resources are not fully shared and utilised, so further collaborative exchanges are needed.

Cocitation analysis of authors can reveal trends in STING signaling pathways and cancer related research. Cocitations are established when two (or more) authors are cited simultaneously in one or more subsequent papers. The CiteSpace software is used to create an author cocitation map. By analyzing the co-occurrence atlas of authors (Figure 3(b)), we can clearly understand the core authors and their contributions to a field, the intensity of which represents the extent of their involvement in that field. Every node in the graph represents an author, the size of circle represents the number of articles published by researchers, and the connecting lines between circles represent the co-occurrence relationships between the authors. As can be seen from the graph, a close collaboration network has been formed between the authors, which shows that the fields of study between the authors are similar and the references are extensive. Ishikawa H (*n* = 522) ranked no. 1 in the top 10 cocited authors (Table 1), followed by Sun LJ (*n* = 439) and Corrales L (*n* = 380). There was a close co-occurrence relationship between the authors and cocited authors, with authors who contributed a high number of publications tending to have higher co-occurrence with others.

### 3.4. Cocited Academic Journals

**Figure 4:**
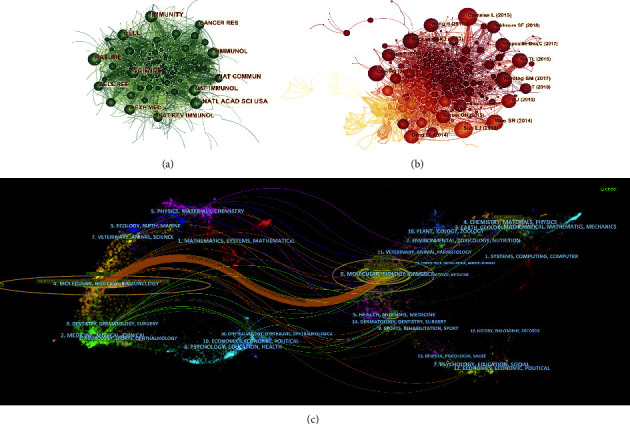
A network map showing cocited academic journals (a) and cocited references (b) involved in the research on STING signaling pathway and cancer. (c) A dual-map overlay of journals related to the research on STING signaling pathway and cancer.

A cocitation network was used to identify the key journals of STING signaling pathways and cancer. The node of the network was set to cited journals, which drew a total of 843 cited journals on STING signaling pathways and cancer (Figure 4(a)), with NATURE (*n* = 1150, IF = 49.962) at the top of the list, followed by SCIENCE (*n* = 1003, IF = 47.728) and PNATL ACAD SCI USA (*n* = 992, IF = 11.205). Among the top ten cited journals (Table 2), 90% (9/10) were from the United States, and the other was from the United Kingdom. In addition, three of the top 10 journals have an impact factor of more than 40, which are: NATURE (IF = 49.962), SCIENCE (IF = 47.728), and CELL (IF = 41.584). As shown in Figure 4(c), there is a positive citation relationship between the different journals.

The double map overlay of journals in Figure 4(c) shows the subject distribution of journals. Citing journals are located on the left side of the map and cited journals are located on the right side, with these labels representing the disciplines covered by the journals. From left to right, coloured lines indicate citation paths. A major citation path indicates that research in MOLECULAR/BIOLOGY/GENETICS journals is frequently cited in MOLECULAR/BIOLOGY/IMMUNOLOGY journals.

A cocitation network was used to analyze the key literature on STING signaling pathway and cancer. The nodes of the network were set to references (citations) and created the literature cocitation network (Figure 4(b)) with a density of 0.0117, 950 nodes, and 5266 connecting lines, indicating that the references are relatively dispersed across citations. Table 2 shows the top 10 references in terms of frequency of citations [17–26]. The paper entitled “Regulation and function of the cGAS-STING pathway of cytosolic DNA sensing” by Chen Q et al. [17] was the most frequently cited (*n* = 208). In addition, two of the top 10 references in terms of citation frequency have the highest impact factors, “cGAS surveillance of micronuclei links genome instability to innate immunity” by Mackenzie et al.[19] et al. and “Mitotic progression following DNA damage enables pattern recognition within micronuclei” by Harding S.M. et al. [20] both with an impact factor of 49.962 and their citations of 180 and 172, respectively.

The top 15 references with the strongest citation bursts were identified via a document cocitation strength analysis, which is a method for determining research trends. “Citation Burst” indicates that a reference has been widely cited over time. References marked in red in the table indicate a sudden increase in the frequency of citations during this period, while blue indicates a period of relative unpopularity.  Table 3 shows the top 15 references with the strongest citation bursts [27–41]. “Kwon J, 2020, CANCER DISCOV, V10, P26, DOI 0.1158/2159-8290. CD-19-0761” [33] is a burst reference in 2021–2022, with a burst strength of 21.08. We can predict that the research directions of Kwon J and others will become hotspots in the next few years and will be cited by more scholars. “Sun LJ, 2013, Science, V339, P786, DOI 10.1126/science. 1232458” [22] has the strongest citation bursts (strength = 62.25) and lasts for six years (2013–2018), which is to say that Sun LJ et al. 's articles have been frequently cited since their publication in 2013, and their popularity lasts until 2018.

### 3.5. Analysis of Keywords

#### 3.5.1. A Network Map of Keywords

**Figure 5:**
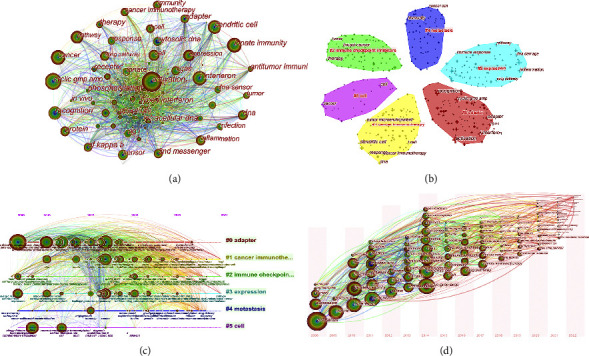
A network map of keywords involved in the research on STING signaling pathway and cancer. (a) Clustered networks of keywords via CiteSpace. The top 6 largest clusters. (b) Timeline view (c) Timezone view (d) of keywords involved in the research on STING signaling pathway and cancer.

Keywords are highly generalizable to the topic and core content of the article. Through network map of keyword analysis, we can understand the distribution and development of different research hotspots in a certain field. In order to further analyze the research hotspots on STING signaling pathway and cancer, a network map of keywords (Figure 5(a)) is constructed, density = 0.0302, *N* = 495, and *E* = 3698. The keywords of the hotspot include activation, STING pathway, cyclic GMP AMP, dendritic cell, I interferon, innate immunity, inflammation, cGAS, T cell, and tumor microenvironment. The high frequency of activation, STING pathway, and immunity indicates that STING signaling pathway is more and more concerned in the field of tumor immunotherapy.

#### 3.5.2. Clustered Networks in Keywords

Then, we performed clustered network analysis to conduct a more in-depth study of those keywords. Cluster analysis is a statistical method to classify the data with multiple indexes and classify the indexes according to the similarity of the indexes. Based on the research of STING signaling pathway and cancer correlation, 6 kinds of clusters were formed by clustering (Figure 5(b)) on the basis of keyword network, density = 0.0302, *N* = 495, *E* = 3698, and weighted mean silhouette = 0.7071. Figure 5(b) shows that the research on STING signaling pathway and cancer are mainly focused on six clusters: #0 adapter (including activation/I-interferon/cGAS), #1 cancer therapy (including microenvironment/dendritic cell/T cell), #2 inhibitors (including treatment/tumor/cancer), #3 (including pathway/immune rspondation/inflammation), #4 metastasis (including cancer/immunity cell), and #5 (including cancer/cell). Simply put the cluster of #0 contains the largest number of articles.

#### 3.5.3. Research Hotspots and Development Trend Analysis

To explore changes in topics related to STING signaling pathways and cancer, the CiteSpace software was used to construct Timeline View [42] (Figure 5(c)), Timezone View [43] (Figure 5(d)), density = 0.0302, *N* = 495, and *E* = 3698. The Timeline View is a way to visualize data that combine clustering and time slicing techniques. Clustering labels are sorted according to the time of appearance, which can not only explain the distribution of topics in the field, but also show the trend of research topics with time. In the Timeline View, the different colours of the nodes on the same row represent different years. The node on the left represents the earlier keyword and the node on the right represents the newer keyword. The same horizontal line indicates the cluster to which the keyword belongs, and the aggregation label is located at the rightmost end of the row. Figure 5(c) shows the evolution of keywords in each cluster over time and the frequency of keywords in the past 15 years. Figure 5(d) shows the keywords that appeared in the year and the interconnections between the different years. The keywords with high frequency of occurrence in the last 5 years can represent the main focus research on STING signaling pathway and cancer.

Keywords with the strongest citation bursts not only can understand the current research hotspot, but also can predict the future development trend of the field. The red line indicates that the use of a keyword increased suddenly during the relevant period, on the contrary, a blue line means relative unpopularity.  Table 4 shows that the word “cytosolic DNA” [29, 44–46] with the strongest burst strength has lasted for seven years since 2011(strength = 13.19). We can infer that during this period, this area has maintained a relatively high level of research heat. The word “cGAS STING pathway” [38, 47–51] is a keyword that has emerged in the last two years (2021–2022) with a strength of 5.52.

## 4. Discussion

Cancer has plagued humans since prehistoric times, and the first written descriptions of human cancer appeared in the ancient Egyptian manuscripts found in the 19th century [52]. Classical antitumor therapies include chemotherapy, radiotherapy, and surgery [35]. However, radiotherapy and chemotherapy have a greater impact on human immunity and some tumors are not sensitive to them. Novel immunotherapy are transforming the treatment of cancer over the past two decades [36, 37]. The cGAS-STING signaling pathway has a dichotomous role in cancer, with both autonomous and involuntary antitumor effects in tumor cells [38]. Song et al. [39] demonstrated that STING protein expression was remarkably decreased in gastric cancer can promote the progression of tumor, promote colony formation, viability, migration, and invasion of gastric cancer cells and STING can be regarded as a potential prognostic marker for gastric cancer patients. The dichotomous role of the cGAS-STING signaling pathway in tumors is that DNA damage induces chronic inflammatory signaling through sustained activation of NF-*κ*B downstream of the cGAS-STING signaling pathway, facilitating epithelial-to-mesenchymal transition (EMT), and leading to increased migration and invasion of cancer cells. After activation of cGAS and effector STING, cytoplasmic DNA stimulates the secretion of interferon beta by cancer cells [53]. Lemos et al. [40] have shown that cytosolic DNA induces and activates STING to promote Lewis lung cancer (LLC) growth. Activation of STING can attenuate tumor cell killing and promote the tolerant response during LLC growth and upregulate IDO expression, directly or indirectly inhibiting T cell number and function, thereby promoting immune escape.

Due to the important role of STING in natural immune modulation, the research on its agonists has also become a hot topic in recent years. Many natural and synthetic STING agonists have entered the clinical development stage and have been tested in preclinical and clinical settings for different tumors [41], which include natural CDNs, CDN derivatives, flavonoids and xanthones, and other novel and unique compounds [54]. DMXAA is the most prominent preclinical and widely used STING agonist, which is a vascular disruptor with known antitumor activity [41]. Immunosuppression is frequently accompanied by the attraction of immunosuppressive [55]. cGAMP is one of the STING agonists, which can activate the STING signaling pathway, boost the innate immune system to activate CD3(+) CD8(+) T cells and related cytokines, and reduce the number of MDSCs in the body. In addition, EMT can be inhibited by Wnt/beta-catenin pathway, thereby inhibiting cancer cell metastasis [56]. It has been demonstrated that STING agonists can inhibit the growth and proliferation of breast cancer cells and can be used not only in the treatment of breast cancer, but also in combination with atenrizumab to enhance the efficacy of immunotherapy [57]. However, persistent overactivation of STING induces chronic inflammation and involves a wide range of autoimmune diseases [38, 41], and whether its activation induces a negative feedback loop that inhibit the action of STING agonists remains to be further investigated.

Based on 1670 core articles on STING signaling pathway and cancer from 123 countries and 406 institutions collected by Web of Science Core Collection database in 2008–2022, CiteSpace software is used to draw the corresponding network map by visual analysis, keyword clustering, and Timeline view. It helps us to analyze the literature of STING signaling pathway and cancer research direction in the last 15 years from multiple dimensions, and comprehensively understand the development process of the field, which can enable us to accurately predict the future development of the field, collect and share valuable research information for scholars who are new to this research field.

The above results show an overall upward trend in articles on STING signaling pathways and cancer between 2008 and 2022. Especially, since 2017, the number of papers published in this field has increased rapidly, reaching a peak of 421 in 2021. Based on this trend, we predict that relevant research in this field will continue to increase in the coming years. Of the 123 countries that have done research in this area globally, the United States has made the highest contribution to the research on STING signaling pathways and cancer because it has cooperated with the rest of the world 919 times, and we can predict that the United States will continue to work with other countries in this area to maintain high yields. Among the top 10 countries in the world, China ranked no. 2, accounting for only 18%, indicating that China has less research on STING signaling pathways and cancer, and that China lacks cooperation with other countries in this field. It is suggested that there is still a lot of room for further research in this field, and more scholars need to devote themselves to this direction for further study and strengthen international cooperation. Nine of the top ten institutions with the most collaborative publications are located in the United States, and these institutions have close collaborative relationships with each other and with other institutions, so it is clear that the U.S. institutions have made the greatest academic contributions in this field. The relationship between the authors forms a close cooperative network, which shows that the research directions of the authors in this field are similar and widely cited from each other. Ishikawa H has a high influence in this field, and its research results have been widely recognized and cited. Glen N. Barber is the most prolific scholar in this field. There are close cooperative relations between Chinese scientific research institutions, especially between Chinese Academy of Sciences and Wuhan University. However, from the perspective of institutional cooperation network, domestic research institutions have less contact with international institutions, which suggest that scholars need to strengthen cooperation with international institutions in the future research.

### 4.1. Research Hotspots

The development trend in this field can be determined by cocitation analysis. The results of Chen et al. [17] were cited most often. Their study mainly described the mechanism of cGAS-STING signaling pathway in inflammation and cancer and found that cGAS-STING can mediate not only protective immune defense but also antitumor immunity. STING is a cytoplasmic receptor that induces type I interferon and proinflammatory cytokine responses by activating TBK1/IRF3, NF-kB, and STAT6 signaling pathways. The antitumor activity depends on STING and is associated with increased activation of dendritic cells and tumor antigen-specific CD8 (+) T cells [20]. Mackenzie et al. [19] concluded that micronuclei are an important source of immunostimulatory DNA, with the highest impact factor. The most recent article with the strongest citation bursts in 2021–2022, published by Kwon et al., demonstrates that cGAS-STING participates in the inhibition or promotion of malignancies in addition to its role in antiviral immunity [33]. Therefore, the relationship between STING signaling pathway and cancer is a hot topic of the research recently, and it will continue to be concerned in this field in the next few years. The Network Map of Keywords, Timeline View, and Timezone View indicate that the association between “STING pathway” and “cancer/tumor” is increasing in recent years. The keyword “cGAS STING Pathway” of the strongest citation bursts from 2021 to 2022 indicates that there is a sharp increase in this research direction, which has a good research space and development prospects. STING agonists can effectively initiate tumor specific CD8 (+) T cell immune response, induce tumor regression, increase T cell expression, and enhance tumor immune response, which is also a new direction of follow-up research on malignant tumors.

## 5. Conclusion

In summary, STING signaling pathway is potential to become a new immune target for clinical prevention and treatment in cancer. In this paper, we summarize the current status and trends of the research on STING signaling pathway and cancer in the past 15 years, and it is clear that it can play an antitumor role in most cancers by activating the body's immune response with specific CD8(+) T cells, but there are less studies on its promotion in tumor progression. It also suggests that we should strengthen our collaboration and conduct more extensive and in-depth research on STING and cancer, providing new ideas for future immunotherapy of malignant tumors. Whether STING agonists can synergise with more conventional radiotherapy and Chinese medicine to achieve greater antitumor efficacy and what role they can play in multidrug resistance in cancer needs to be further explored.

## Figures and Tables

**Table 1:** The top 10 cocited authors and the top10 authors with the most published papers involved in the research on STING signaling pathway and cancer.

Cited authors	Count	Year	Authors	Count	Year
Ishikawa H	522	2009	Glen N Barber	48	2008
Sun LJ	439	2013	Zhijian J Chen	23	2012
Corrales L	380	2015	Leticia Corrales	17	2015
Woo SR	363	2015	Sarah M McWhirter	14	2015
Ablasser A	356	2009	Thomas F Gajewski	13	2014
Chen Q	290	2017	Jeonghyun Ahn	12	2013
Burdette DL	276	2012	David B Kanne	12	2015
Deng LF	272	2015	Thomas W	12	2015
Wu JX	268	2013	Hiroyasu Konno	11	2010
Ahn J	265	2013	Yangxin Fu	11	2017

**Table 2:** The top 10 cocited academic journals and the top 10 cocited references related to the research on STING signaling pathway and cancer.

Rank	Journal	IF	Cocited reference	IF
1	NATURE	49.962	Chen Q, 2016, NAT IMMUNOL, V17, P1142, DOI 10.1038/ni.3558	25.606
2	SCIENCE	47.728	Corrales L, 2015, CELL REP, V11, P1018, DOI 10.1016/j.celrep.2015.04.031	9.423
3	P NATL ACAD SCI USA	11.205	Mackenzie KJ, 2017, NATURE, V548, P461, DOI 10.1038/nature23449	49.962
4	IMMUNITY	31.745	Harding SM, 2017, NATURE, V548, P466, DOI 10.1038/nature23470	49.962
5	CELL	41.584	Woo SR, 2014, IMMUNITY, V41, P830, DOI 10.1016/j.immuni.2014.10.017	31.745
6	J IMMUNOL	5.422	Sun LJ, 2013, SCIENCE, V339, P786, DOI 10.1126/science.1232458	47.728
7	CELL REP	9.423	Deng LF, 2014, IMMUNITY, V41, P843, DOI 10.1016/j.immuni.2014.10.019	31.745
8	NAT IMMUNOL	25.606	Fu J, 2015, SCI TRANSL MED, V7, P0, DOI 10.1126/scitranslmed.aaa4306	17.956
9	NAT COMMUN	14.919	Li T, 2018, J EXP MED, V215, P1287, DOI 10.1084/jem.20180139	14.307
10	CANCER RES	12.701	Wang H, 2017, P NATL ACAD SCI USA, V114, P1637, DOI 10.1073/pnas.1621363114	11.205

**Table 3:** The top 15 references with the strongest citation bursts. References marked in red indicates a sudden increase in cited frequency of this article during that period. Blue represents a relatively unpopular time period.

References	Strength	Begin	End	2008–2022
Ishikawa H, 2009, NATURE, V461, P788, DOI 10.1038/nature08476	29.98	2011	2014	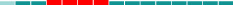
Unterholzner L, 2010, NAT IMMUNOL, V11, P997, DOI 10.1038/ni.1932	24.74	2011	2015	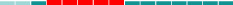
Burdette DL, 2011, NATURE, V478, P515, DOI 10.1038/nature10429	40.96	2012	2016	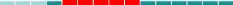
Sun LJ, 2013, SCIENCE, V339, P786, DOI 10.1126/science.1232458	62.25	2013	2018	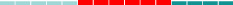
Wu JX, 2013, SCIENCE, V339, P826, DOI 10.1126/science.1229963	35.92	2013	2018	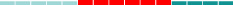
Ablasser A, 2013, NATURE, V498, P380, DOI 10.1038/nature12306	27.34	2013	2018	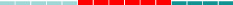
Ahn J, 2012, P NATL ACAD SCI USA, V109, P19386, DOI 10.1073/pnas.1215006109	24.44	2013	2017	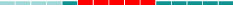
Zhang X, 2013, MOL CELL, V51, P226, DOI 10.1016/j.molcel.2013.05.022	24.37	2013	2018	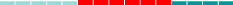
Tanaka Y, 2012, SCI SIGNAL, V5, P0, DOI 10.1126/scisignal.2002521	22.59	2013	2017	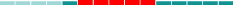
Diner EJ, 2013, CELL REP, V3, P1355, DOI 10.1016/j.celrep.2013.05.009	21.03	2013	2018	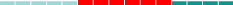
Li XD, 2013, SCIENCE, V341, P1390, DOI 10.1126/science.1244040	23.42	2014	2018	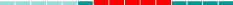
Konno H, 2013, CELL, V155, P688, DOI 10.1016/j.cell.2013.09.049	21.1	2014	2018	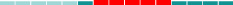
Deng LF, 2014, IMMUNITY, V41, P843, DOI 10.1016/j.immuni.2014.10.019	35.63	2015	2019	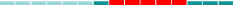
Woo SR, 2014, IMMUNITY, V41, P830, DOI 10.1016/j.immuni.2014.10.017	43.99	2016	2019	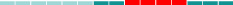
Kwon J, 2020, CANCER DISCOV, V10, P26, DOI 10.1158/2159–8290.CD-19-0761	21.08	2021	2022	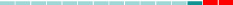

**Table 4:** The top 15 keywords with the strongest citation bursts. A keyword marked red indicates a sudden increase in the frequency of use of the keyword during this period. Blue represents a relatively unpopular period.

Keywords	Strength	Begin	End	2008–2022
Adapter protein	5.59	2009	2013	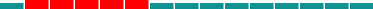
Cytosolic DNA	13.19	2011	2017	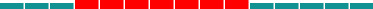
Recognition	10.9	2011	2016	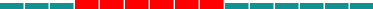
Intracellular DNA	8.67	2011	2017	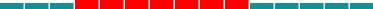
NF kappa B	4.57	2011	2014	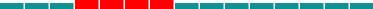
Sensor	6.27	2012	2016	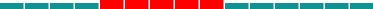
Cyclic di GMP	10.74	2013	2017	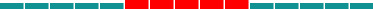
Innate immune sensor	5.73	2013	2016	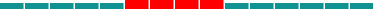
Innate immune response	5.2	2013	2017	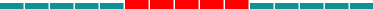
Interferon	4.96	2013	2017	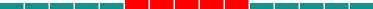
Infection	4.63	2014	2018	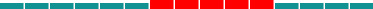
Immunogenic tumor	8.39	2015	2018	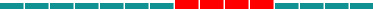
Cytosolic DNA sensor	5.83	2015	2017	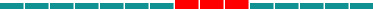
Cyclic GMP AMP	6.32	2016	2017	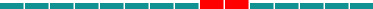
cGAS STING pathway	5.52	2021	2022	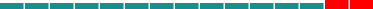

## Data Availability

The [plain text format] data used to support the findings of this study are available from the corresponding author upon request.
